# Comment on “Bayesian additional evidence for decision making under small sample uncertainty”

**DOI:** 10.1186/s12874-022-01635-4

**Published:** 2022-05-25

**Authors:** Samuel Pawel, Leonhard Held, Robert Matthews

**Affiliations:** 1 Department of Biostatistics, University of Zurich, Zurich, Switzerland; 2 Department of Mathematics, Aston University, Birmingham, UK

**Keywords:** Reverse-Bayes, Analysis of credibility, Bayesian additional evidence, Advocacy prior

## Abstract

We examine the concept of Bayesian Additional Evidence (BAE) recently proposed by Sondhi et al. We derive simple closed-form expressions for BAE and compare its properties with other methods for assessing findings in the light of new evidence. We find that while BAE is easy to apply, it lacks both a compelling rationale and clarity of use needed for reliable decision-making.

## Introduction

We read with great interest the article by Sondhi et al. [1], which introduces the concept of *Bayesian Additional Evidence* (BAE). The authors use a reverse-Bayes argument to define BAE, and apply it to the important issue of how new evidence affects the overall credibility of an existing finding. As they state, BAE is thus closely related to another reverse-Bayes approach known as *Analysis of Credibility* (AnCred) proposed by Matthews [2]; see also the recent review of Reverse-Bayes methods [3]. In what follows, we comment on the similarities and differences of the two approaches and their inferential consequences. We find that decision making based on the BAE approach is limited by the restrictive assumption that the additional evidence must have equal or smaller variance than the variance of the observed data.

## Bayesian additional evidence

We begin by showing that fortunately – and contrary to the statement by Sondhi et al. on page 4 of their article – there is a closed-form solution for what they term the BAE “tipping point”, which is key to their approach.

Assume, as per Sondhi et al., that both the likelihood of an effect estimate $\hat {\theta }$ (the “data”) and the prior of the underlying effect size *θ* are represented by normal distributions $\hat {\theta }\,\vert \, \theta \sim \mathrm {N}(\theta, \sigma ^{2})$ and *θ*∼N(*μ*,*τ*^2^), with the latter evidence coming either from pre-existing insight/studies or from a subsequent replication. Bayes’s Theorem then implies a posterior distribution $\theta \,\vert \, \hat {\theta } \sim \mathrm {N}(\mu _{p}, \tau ^{2}_{p})$ whose mean and variance satisfy

$$\begin{array}{*{20}l} &\frac{\mu_{p}}{\tau^{2}_{p}} = \frac{\hat{\theta}}{\sigma^{2}} + \frac{\mu}{\tau^{2}}& &\text{and}& &\frac{1}{\tau^{2}_{p}} = \frac{1}{\sigma^{2}} + \frac{1}{\tau^{2}}&\end{array} $$

Sondhi et al. further assume that *τ*^2^=*σ*^2^, that is, the prior variance *τ*^2^ is equal to the data variance *σ*^2^ which itself is equal to the squared (known) standard error *σ* of the effect estimate $\hat {\theta }$. It then follows that the posterior mean is the mean of the data and the prior mean, and that the posterior variance is half the data variance

1 $$\begin{array}{*{20}l} &\mu_{p} = \frac{\hat{\theta} + \mu}{2}& &\text{and}& &\tau^{2}_{p} = \frac{\sigma^{2}}{2}&  \end{array} $$

The BAE “tipping point” is then defined as the least extreme prior mean that results in a posterior credible interval which excludes the null value. If the substantive hypothesis is for positive effect estimates (*e.g.* log(HR)>0) the BAE is the prior mean which leads to the lower limit *L*_*p*_ of the 100(1−*α*)% posterior credible interval being zero

2 $$\begin{array}{*{20}l} L_{p} = \mu_{p} - z_{\scriptscriptstyle \alpha/2} \, \tau_{p} = 0  \end{array} $$

while for negative effect estimates the upper limit *U*_*p*_ is fixed to zero

3 $$\begin{array}{*{20}l} U_{p} = \mu_{p} + z_{\scriptscriptstyle \alpha/2} \, \tau_{p} = 0  \end{array} $$

with *z*_*α*/2_ the 1−*α*/2 quantile of the standard normal distribution. Combining Eq. (1) with Eq. (2), respectively Eq. (3), leads to

4 $$\begin{array}{*{20}l} \text{BAE} & = \text{sign}(\hat{\theta}) \sqrt{2} \, z_{\scriptscriptstyle \alpha/2} \, \sigma - \hat{\theta}  \end{array} $$

where $\text {sign}(\hat {\theta }) = 1$ when $\hat {\theta } > 0$ and $\text {sign}(\hat {\theta }) = -1$ otherwise. Re-written in terms of the upper and lower 100(1−*α*)% confidence interval (CI) limits *U* and *L* of the effect estimate $\hat {\theta }$ we obtain

5 $$\begin{array}{*{20}l} \text{BAE} = \frac{\text{sign}(\hat{\theta}) \sqrt{2} (U - L) - (U + L)}{2}  \end{array} $$

We see from Eq. (4) that Sondhi et al.’s proposal has the intuitive property that as the study becomes more convincing (through larger effect sizes $|\hat {\theta }|$ and/or smaller standard errors *σ*), the BAE will decrease (increase) for positive (negative) $\hat {\theta }$, indicating that less additional evidence is needed to push a non-significant study towards credibility. Eq. (4) and Eq. (5) also hold for significant studies but the BAE then represents the mean of a “sceptical” prior which renders the study non-significant.

**Fig. 1:**
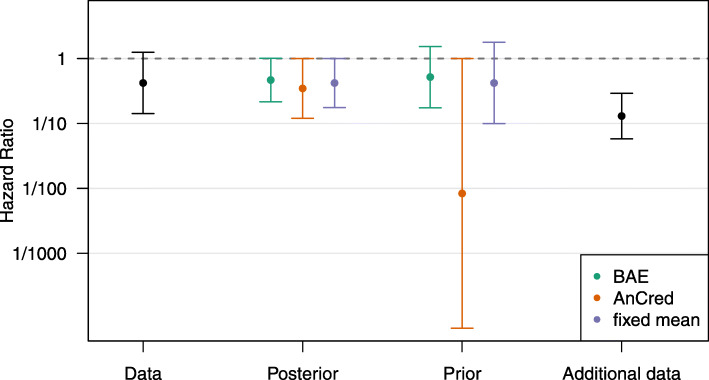
Comparison of BAE, AnCred, and fixed mean 95% prior and posterior credible intervals for the data from Sondhi et al. [1]. Additional data from Innocenti et al. [4] are also shown

These closed-form solutions greatly simplify the use of the BAE methodology. For example, Sondhi et al. use a comparison of monoclonals to show how it identifies additional evidence which, when combined with a non-significant finding, leads to overall credibility. The trial estimated the hazard ratio of the bevacizumab+chemo patients compared to the cetuximab+chemo patients as HR=0.42 (95% CI: 0.14 to 1.23), a non-significant finding with *p*=0.11. Expressed as log(HR), we have *L*=−1.97 and *U*=0.21. We use Eq. (5) and find that on log hazard ratio scale BAE=−0.66 equivalent to an HR of 0.52. Figure 1 shows the corresponding prior mean with 95% prior credible interval.

Thus additional evidence in the form of prior insight or a subsequent replication supporting an HR at least as impressive as this (*i.e.* an HR<0.52 in this case), and a CI at least as tight as that of the original study will render this non-significant result credible at the 95% level. Sondhi et al. cite prior evidence from Innocenti et al. [4] who found an HR=0.13 (95% CI: 0.06 to 0.30) which meets both criteria set by the BAE, and renders the original study credible.

## Alternatives approaches

In order to get a unique solution for the BAE, Sondhi et al. make the assumption that the prior variance equals the data variance, but also other possibilities exist. An alternative rationale would be to set the mean of the additional evidence, rather than variance, to that of the original finding (*i.e.*$\mu = \hat {\theta }$), and determining the prior variance *τ*^2^ such that the posterior credible interval includes the null value. Under this approach, the prior variance is given by

$$\begin{array}{*{20}l} \tau^{2} = \frac{\sigma^{2}}{z_{\scriptscriptstyle \alpha/2}^{2}/z^{2} - 1} \end{array} $$

with $z = \hat {\theta }/\sigma $. The resulting prior represents a study with identical effect estimate but different precision compared to the observed one. As the observed study becomes more convincing (with larger effect estimates $|\hat {\theta }|$ and/or smaller standard errors *σ*), the prior will become more diffuse, so less additional evidence is needed to render the finding credible. We see in Fig. 1 that prior and posterior are similar to BAE for the clinical trial data from Sondhi et al.

Figure 1 also illustrates that the BAE and the fixed mean approach can lead to priors which support effect sizes opposing that of the original finding. This is not possible with the AnCred advocacy prior whose prior credible interval is fixed to the null value so that the prior adheres to the Principle of Fairminded Advocacy [2]. Held et al. [3] showed that this constraint is equivalent to fixing the coefficient of variation from the prior to $\tau /\mu = z_{\scriptscriptstyle \alpha /2}^{-1}$. Hence, its mean and variance are given by

$$\begin{array}{*{20}l} &\mu = \frac{2 \, \hat{\theta}}{1 - z^{2}/z_{\scriptscriptstyle \alpha/2}^{2}}& &\text{and}& &\tau^{2} = \frac{\mu^{2}}{z_{\scriptscriptstyle \alpha/2}^{2}}.& \end{array} $$

We see that – as with the fixed mean approach – the AnCred prior becomes more diffuse for increasingly convincing studies. However, at the same time the prior mean also increases (decreases) for positive (negative) effect estimates, so that only effect sizes in the correct direction are supported.

Figure 1 shows that the AnCred advocacy prior credible interval is far wider compared to the other approaches. Perhaps this observation led Sondhi et al. to state that AnCred is harder to interpret than BAE, and that it can lead to prior intervals “wide enough to effectively contain any effect size, which is unhelpful for decision making”. We would argue that broad priors are a valuable diagnostic of when little additional evidence is needed to achieve posterior credibility, as it is the case with the example Sondhi et al. consider. Moreover, we would argue that AnCred priors are very helpful in decision making since any additional evidence whose confidence interval is contained in the AnCred prior credible interval will *necessarily* lead to posterior credibility when combined with the observed data [3]. In contrast, the BAE approach requires decision makers to keep in mind the variance of the additional evidence, since only additional evidence with a point estimate that is at least as extreme as the BAE and with confidence interval at least as tight as the observed confidence interval from the study is guaranteed to lead to posterior credibility. Assume, for example, the additional data from Innocenti et al. had been more impressive, say, HR=0.05, with a 95% CI from 0.015 to 0.16. Intuition suggests, and direct calculation confirms, that this would be even more capable of making the original finding credible. However, this would not be clear to a decision maker using the BAE approach as currently formulated, as the confidence interval is wider than the one of the observed study (on the log scale).

**Fig. 2:**
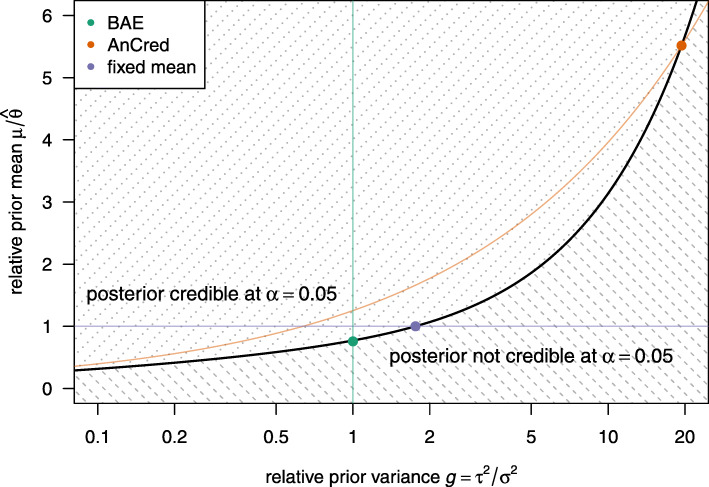
Relative prior mean vs. relative prior variance for the data from Sondhi et al. The dashed region represents parameter values, which do not lead to posterior credibility, whereas values in the dotted region lead to posterior credibility (at *α*=5*%*). The colored lines indicate the parameters which fulfil the side-constraints of the respective method

While Sondhi et al. acknowledge the dependence of the BAE on the choice of the prior variance, they do not give clear guidance on when it should be set to a value different from the observed data variance. Fortunately, when the prior and data variances differ, there is again a closed form solution for the BAE “tipping point”

6 $$\begin{array}{*{20}l} \text{BAE}(g) & = \text{sign}(\hat{\theta}) \sqrt{g \, (1 + g)} \, z_{\scriptscriptstyle \alpha/2} \, \sigma - g \, \hat{\theta}  \end{array} $$

with relative prior variance *g*=*τ*^2^/*σ*^2^. We see from Fig. 2 that Eq. (6) substantially depends on the chosen prior variance and that the BAE based on *g*=1 only captures a limited range of priors which lead to posterior credibility. Unfortunately, Sondhi et al. do not give a clear rationale for the default choice of *g*=1. It may therefore be more helpful for decision makers to base their decision on the more principled AnCred advocacy prior or on a visualisation of the prior parameter space as in Fig. 2.

## Conclusion

In summary, we welcome BAE as an interesting application of reverse-Bayes methods, and we hope our derivation of closed-form solutions will encourage further research. However, as currently formulated BAE lacks both a clear rationale for the constraints on which it is based, and a sufficiently detailed explanation allowing reliable decision-making.

## Data Availability

Summary statistics on the case study were taken from Sondi et al. The R Code to reproduce our analyses is available on the Open Science Framework (https://osf.io/ymx92/).

## Abbreviations

BAE: Bayesian additional evidence
AnCred: Analysis of credibility
